# Dietary protein sources differentially affect microbiota, mTOR activity and transcription of mTOR signaling pathways in the small intestine

**DOI:** 10.1371/journal.pone.0188282

**Published:** 2017-11-17

**Authors:** Soumya K. Kar, Alfons J. M. Jansman, Nirupama Benis, Javier Ramiro-Garcia, Dirkjan Schokker, Leo Kruijt, Ellen H. Stolte, Johanna J. Taverne-Thiele, Mari A. Smits, Jerry M. Wells

**Affiliations:** 1 Host Microbe Interactomics Group, Wageningen University & Research, Wageningen, the Netherlands; 2 Animal Breeding and Genomics Centre, Wageningen University & Research, Wageningen, the Netherlands; 3 Wageningen Livestock Research, Wageningen University & Research, Wageningen, the Netherlands; 4 Laboratory of Microbiology, Wageningen University & Research, Wageningen, the Netherlands; 5 Top Institute Food and Nutrition, Wageningen, the Netherlands; 6 Laboratory of System and Synthetic Biology, Wageningen University & Research, Wageningen, the Netherlands; 7 Wageningen Bioveterinary Research, Wageningen University & Research, Wageningen, the Netherlands; University of Illinois at Urbana-Champaign, UNITED STATES

## Abstract

Dietary protein sources can have profound effects on host-microbe interactions in the gut that are critically important for immune resilience. However more knowledge is needed to assess the impact of different protein sources on gut and animal health. Thirty-six wildtype male C57BL/6J mice of 35 d age (n = 6/group; mean ± SEM body weight 21.9 ± 0.25 g) were randomly assigned to groups fed for four weeks with semi synthetic diets prepared with one of the following protein sources containing (300 g/kg as fed basis): soybean meal (SBM), casein, partially delactosed whey powder, spray dried plasma protein, wheat gluten meal and yellow meal worm. At the end of the experiment, mice were sacrificed to collect ileal tissue to acquire gene expression data, and mammalian (mechanistic) target of rapamycin (mTOR) activity, ileal digesta to study changes in microbiota and serum to measure cytokines and chemokines. By genome-wide transcriptome analysis, we identified fourteen high level regulatory genes that are strongly affected in SBM-fed mice compared to the other experimental groups. They mostly related to the mTOR pathway. In addition, an increased (*P* < 0.05) concentration of granulocyte colony-stimulating factor was observed in serum of SBM-fed mice compared to other dietary groups. Moreover, by 16S rRNA sequencing, we observed that SBM-fed mice had higher (*P* < 0.05) abundances of *Bacteroidales* family *S24-7*, compared to the other dietary groups. We showed that measurements of genome-wide expression and microbiota composition in the mouse ileum reveal divergent responses to diets containing different protein sources, in particular for a diet based on SBM.

## Introduction

For economical reasons soybean meal (SBM) is commonly used as a protein source in animal feeds [1] but the increasing demand and price for SBM is stimulating interest in alternative sources of protein. Apart from the predicted nutritional properties of these novel protein sources, nothing is known about their potential effects on gut immunity and health. The effects of dietary protein on performance and health are dependent on the source(s) of protein (e.g. milk; plasma; plant cereals, insects, algae) included in the diet, their digestibility in the gastro-intestinal tract, the matrix in which the protein fraction is incorporated and the nature and extent of technical processing. Furthermore, the amino acid composition and sequences of the individual proteins may influence the kinetics of protein digestion and the generation of bioactive peptides along the gastro-intestinal tract (GIT) [2–5]. In addition, variations in the non-protein components of protein sources may have direct effects on the health and performance of hosts or indirect effects via modulation of the intestinal microbiota [6]. Alternative sources of protein include dietary casein (CAS) and whey proteins which are reported to stimulate the immune system and promote host protection against allergies [7, 8]. Spray dried plasma protein (SDPP), described as a high-quality feed ingredient for farm animals, including pigs, which is suggested to support intestinal immunity due to the presence of a high amount immunoglobulins [9]. Soybean meal (SBM) is the common source of dietary protein for many mammalian species and contains complex mixture of proteins, carbohydrates such as non-starch polysaccharides (NSP) and phytochemicals, which might influence the activity of the immune system [6]. Wheat gluten meal (WGM) is also considered as a good source of dietary proteins for many mammals, as it is highly digestible and regarded as an excellent source of glutamine and glutamic acid, amino acids which are known to play a role in the modulation of gut immunity [10]. Based on protein digestibility and amino acid composition, yellow meal worm (YMW) seems to be a promising source of dietary protein for human and monogastric animals [11].

Amino acid availability is known to regulate the activity of the mechanistic target of rapamycin (mTOR), pathway which is a key integrator of nutrient, energy and metabolic status, controlling diverse function in different cells types through two protein complexes. Complex 1 (mTORC1) controls cell growth and cell size by phosphorylation of the regulators of protein synthesis S6K1 and 4EBP1, while mTORC2 regulates cell proliferation by functioning as the regulatory kinase [12]. The activity of mTORC1 but not mTORC2 is controlled by nutrient availability whereas both respond to growth factors. The regulation of mTORC1 is better understood than the regulation of mTORC2. Low amino acid availability and energy levels (i.e. low ATP/ADP ratio) inhibit mTOR complex 1 (mTORC1) activity and upregulation of autophagy to recycle nutrients. For example, mTORC1 can sense leucine through leucyl-tRNA synthetase and glutamine levels. Conversely, in the presence of sufficient nutrients, mTORC1 is active, enabling new protein synthesis and cellular growth and proliferation. Diverse pathways can participate in the regulation of mTORC1 signaling under conditions of nutrient availability, including growth factors, cytokines and in T cells signaling via the T cell receptor or co-stimulatory receptor interactions. Thus, mTORC1 can also be seen as a multifaceted regulator of immunity, having a crucial role in the activation and proliferation of effector and regulatory T cells as well as other immune cells [3]. The impact of diet on mTOR signaling in the intestine is not fully understood, not only with regard to the effector pathways, but also with respect to mTOR sensing of novel nutritional and microbial metabolites.

To gain further insights into the effect of different conventional and unconventional dietary protein sources on intestinal energy levels *in vivo*, we performed a transcriptomics analysis and measured mTORC1 protein in tissue. Additionally, we investigated possible effects of the different diets on the microbiota and measured several protein mediators of immunity, inflammation and hematopoiesis in the blood. We deliberately chose to study the effect of different dietary proteins in complex diets because it is known that purified bioactive compounds can behave differently when included in complex dietary mixtures [13, 14]. The experimental diets were prepared by replacing the corresponding reference ingredient (i.e. SBM) from the “reference” diet (i.e. SBM-diet) with CAS, partially delactosed whey powder (DWP), WGM, SDPP and YMW as sources of protein. Mice were fed these experimental diets for a period of four weeks, after which transcriptomics, histology and mTORC1 activity assays were performed on ileal tissue. Additionally ileal digesta were collected for analysis of microbiota by16S rRNA gene sequencing.

## Materials and methods

### Animals

All procedures were approved by the animal experimentation board at Wageningen University & Research Center (accession number 2012062.c) and carried out according to the guidelines of the European Council Directive 86/609/EEC dated November, 1986. A schematic representation and detailed description of the experimental design and sample collection is given in online supporting information (see S1 Fig). Briefly, thirty-six 21-day-old wild type male C57BL/6J mice (Harlan Laboratories, Horst, the Netherlands) were stratified according to bodyweight and litter of origin into 6 diet groups (n = 6/group) in the light and temperature-controlled animal facility of Wageningen University (12:12 h reversed light/dark cycle, 20 ± 2°C) upon arrival. The mice were housed in pairs in a specific pathogen-free environment with ad libitum access to diet and water. Prior to the start of the experiment mice were adapted for one week to a standard diet based on AIN-1993 growth (AIN-93G), which included 300 g/kg casein (CAS) as the protein source (as fed basis). Thereafter, one group continued with the CAS-fed diet, and the other five groups received similar semi-synthetic diets containing 300 g/kg (as fed basis) of one of the alternative protein sources (SBM, DWP, WGM, SDPP or YMW) for 28 days. Body weights of animals were measured every week. Thereafter, the animals were anaesthetized with isoflurane and sacrificed to collect samples. From the same location three segments of ileal tissue were taken, one was snap frozen in liquid nitrogen and stored at -80 C for gene expression studies, the second was fixed with methanol-carnoy’s fixative for immunohistochemistry, and the third tissue segment along with luminal content was collected to perform a community-scale analysis of gut microbiota. Blood samples were collected by orbital puncture and serum was extracted using 500 μl SST tubes (Becton Dickinson, Franklin Lakes, New Jersey) within 30 minutes after collection of the blood. The sera were stored at -20°C for further analysis of cytokine levels. Soybean meal (SBM) diet served as reference to make comparisons with other experimental diets for all analysis, as it is the most widely used protein source in animal feeds [15, 16].

### Diets

Customized semi-synthetic diets based on AIN-93G were prepared replacing casein with any one of five other protein sources *i*.*e*. SBM, DWP, WGM, SDPP and YMW at an inclusion level of 300 g/kg. Representative samples of dried and ground diets were chemically analyzed for dry matter (DM; NEN-ISO 6496 by 4 hours drying at 104°C), nitrogen (N; NEN-ISO 5983–2 by Kjeldahl method and crude protein calculated as Nx6.25), ash (NEN-ISO 5984 after 3 hours ashing at 550°C), ether extract (EE, NEN-ISO 6492 by extraction with petroleum ether) and gross energy (GE; NEN- EN-ISO 9831 by bomb calorimetry). Ingredient and chemical compositions of the experimental diets are presented in supporting information (S1 Table).

### Gene expression

Total RNA extraction from ileal tissue samples, labelling, hybridization of individual samples on Affymetrix GeneChip mouse gene 1.1 ST arrays (Affymetrix, Santa Clara, CA, USA), scanning, quality control and normalization of the resulting datasets was performed as described previously [17] and the data is available in the Gene Expression Omnibus from NCBI with the accession number GSE84442. The output was used for Gene Set Enrichment Analysis (GSEA) [18] with human official gene symbols in which each experimental diet was compared to the diet containing SBM with permutations on gene sets. InteractiVenn [19] was used to visualize significant GSEA results (FDR < 0.05). Subsequently, we defined a set of common core genes, *i*.*e*. the genes that are enriched in the significant differential gene-sets common to all five comparisons. The common core genes were used to build two types of networks in Cytoscape [20]: a network of GO terms restricted to terms with FDR < 0.001 (using the app BINGO) [21]; and a Functional Interaction (FI) network (using the app Reactome FI) [22]. In the BiNGO network, nodes are GO terms and edges are relations between them, the network was restricted to terms with FDR < 0.001. The FI network has genes as nodes and the edges are interactions between the genes (from literature or predictions). FI Nodes with a degree (number of directly connected nodes) greater than 20 were considered as hubs.

### Microbiota

DNA was isolated from snap frozen intestinal segments and the bacterial 16S rRNA V3 region was sequenced by targeted-amplicon 16S sequencing on a Illumina Mi-Seq sequences as previously described [23]. The 16S rRNA gene sequencing reads were analyzed using an in-house pipeline [24]. Shortly, paired-end libraries were filtered to contain only sequence read pairs with perfectly matching primer and barcodes. Resulting sequence reads were separated by sample using the barcodes and operational taxonomic units (OTUs) were assigned using an open reference database and a customized SILVA 16S rRNA reference database [25]. Microbial composition was generated using a workflow based on quantitative insights into microbial ecology (QIIME) v1.2 [26]. The microbial groups that had a *P* < 0.05 in one of the diets vs SBM were considered significant.

### Immunoassay and immunohistochemistry

Endogenous levels of phosphorylated mTOR (Ser2448) was carried on snap frozen intestinal (ileal) segments using a commercial kit (Phospho-mTOR, Cisbio, USA), following manufacturer’s instructions. Briefly, snap frozen tissues were thawed on ice and chopped into small pieces (about 1–3 mm3) using a sharp razor and then washed twice with 5 ml ice-cold phosphate buffer solution (PBS) using a mechanical rotation for 15 mins followed by centrifugation at 720 rpm, for 5 mins at 4°C. Tissue homogenates were prepared in 5 ml conical tubes (Eppendrof, NL) containing 1 ml of ice-cold PBS using a hand-held homogenizer (Turrax, IKA, USA). The homogenised tissue slurry was then passed through cell strainer of mesh size 70 μm (Corning, NL) and the flow through collected in 1.5 ml tubes (Eppendrof, NL). The flow through was centrifuged at 1200 rpm for 5 mins at 4°C to obtain a pellet of cells which was resuspended in 1 ml of ice cold PBS and the cells counted using a haemocytometer. As instructed by the manufacturer, 33,000 cells were added per well in a 384 well plate for the mTORC1 phosphorylation assay. For the assay, along with the samples, we included positive control (supplied in the kit as control lysate) and negative control (added PBS without disintegrated cells; the negative control was used to check the non-specific signal). Using SpectraMax M5e (Molecular Devices, USA), the signals in the 384 wells plate were measured in two different wavelengths i.e. 665 and 620 nm, as recommended by the manufacturer.

For presence of mTOR protein, paraffin sections (5 μm) of fixed tissue were attached to poly-L- lysine-coated glass slides (Thermo Scientific, Germany). Sections were heated for 20 minutes in 0.01 M sodium citrate (pH 6.0) at 100°C, washed 2 times for 15 minutes with TRIS-Buffered Saline-triton (TBS-t) and then incubated for 30 minutes at room temperature in 5% (v/v) rabbit serum (Invitrogen, Life technologies Ltd, Paisley, UK) in TBS. The mTOR protein was detected by incubating the sections with anti-mTOR antibody (Abcam, Cambridge, UK) diluted 1:500 in TBS-t, overnight at 4°C, washing 2 times for 10 minutes with TBS-t and incubation with secondary anti-body goat-anti-rabbit-biotin (Invitrogen, Life technologies Ltd, Paisley, UK) for 60 minutes at room temperature. Avidin-HRP diluted 1:200 in TBS-t was used and kept for 60 minutes at room temperature to detect the secondary antibody. Thereafter the sections were washed for 10 minutes with two changes in TBS-t followed by 10 minutes rinsing in TRIS-HCl buffer (pH 7.6) and incubated with diamino-benzidine. Finally, the slides were immersed in hematoxylin (1:1) and then immediately rinsed under running tap water for 10 minutes. Digital images of transverse sections of the ileum were used to enumerate the positive reaction in the image.

### Cytokine and chemokine profiles

Serum cytokine and chemokine concentrations (pg/ml) were measured using a Bio-Rad Mouse 23-plex kit (Bio-Rad, Hercules, CA, USA). Calibration curves from recombinant cytokine and chemokine standards were prepared for the 8-point standard dilution set with 4-fold dilution steps in sterile PBS. The samples were measured using a Bio-Plex MagPix Multiplex Reader (Bio-Rad Laboratories Inc. by the Luminex Corporation, The Netherlands). The Bio-Plex Manager software's five-parameter logistic curve fitting (5PL) method was used for raw data analysis and calculation of cytokine concentrations.

### Statistical analysis

Results of immunoassay, TLR assay and cytokines are presented as means ± SEM. Statistical analysis was performed by one-way ANOVA followed by post hoc test (Dunnett test: compared all treatment vs. SBM group as control) using GraphPad prism version 5.03 for Windows Vista (GraphPad Software, San Diego, California, USA). *P* value < 0.05 was considered significant.

## Results

### Feed composition and intake

The experimental diets were based on the nutrient requirements provided in the standardised AIN-93G mouse diet but varied somewhat in nutrient composition, for crude protein, crude fat, sugar, NSP, Ca, P, K, Na, Cl and electrolyte balance (S1 Table). At the start of the dietary intervention (age 28 days), mean body weights of other experimental groups were not significantly different compared with the SBM group. From day 42 to 56, mean body weight of the DWP-fed group was significantly less than mice fed the other diets (S2 Fig). Strikingly, in the YMW-fed group, we measured a significantly lower feed intake compared to the SBM fed group (S2 Fig), while the body weight over the experimental period was not significantly different to the other dietary groups. All animals appeared to be healthy throughout the 4 weeks of dietary change.

### Effect of protein source on ileal gene expression

**Fig 1:**
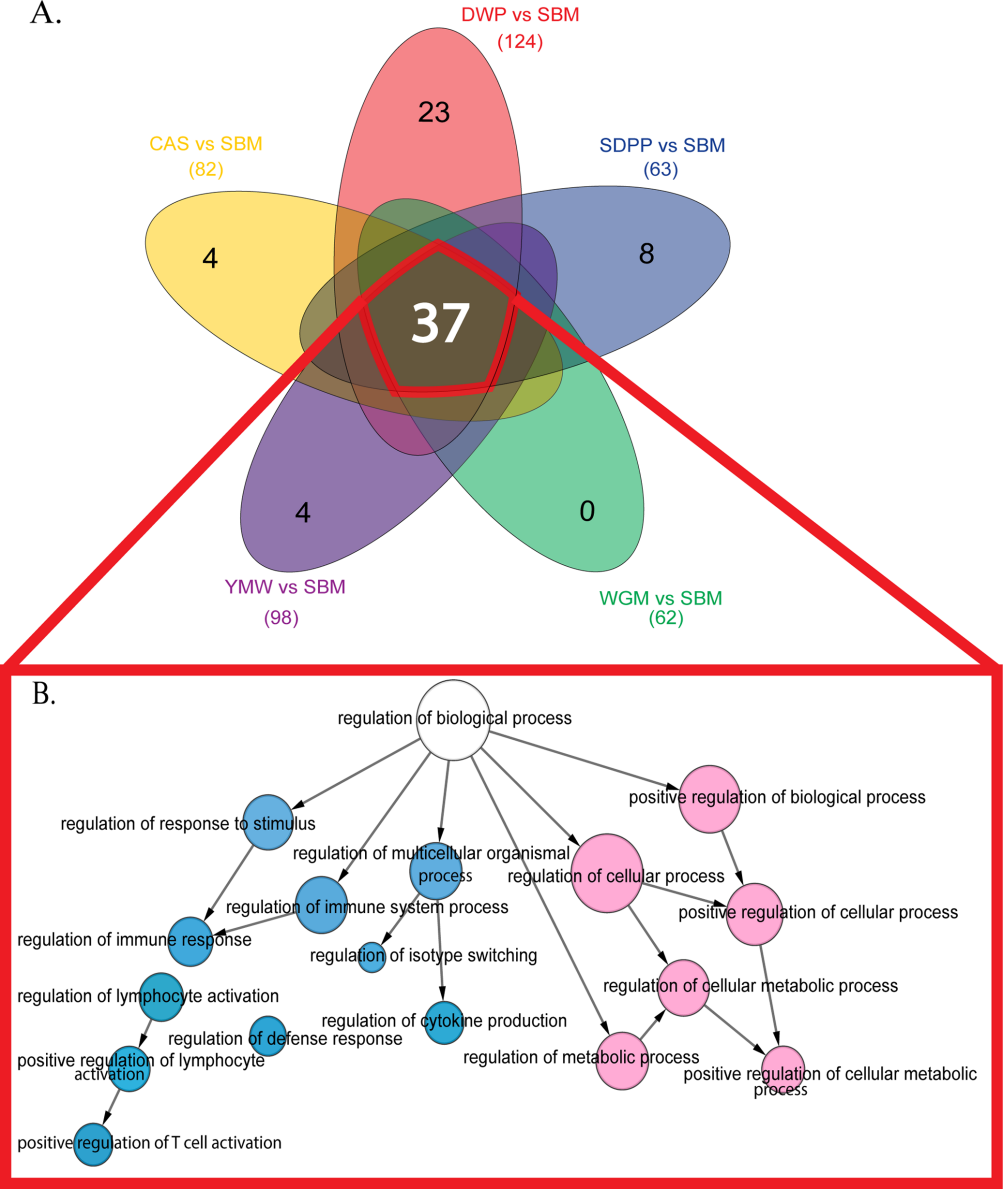
Venn diagram featuring common and unique enriched gene-set expression patterns. (A) Colored spheres represent different experimental diets. The white number at the core of the Venn diagram represents the number of common overlapping gene-sets and black numbers towards the periphery of each sphere represent the number of unique gene-sets. (B) Functional interaction network for the common overlapping gene-sets. The colored nodes denote the GO term significantly (FDR < 0.001) overrepresented in the enriched gene-sets. The edges represent interactions between gene-set as determined by BiNGO. Sky blue color nodes relate to immune processes and light pink color nodes relate to metabolic processes. Arrows represent directed interactions. The diameter of the nodes represents the number of genes associated with that particular GO term. SBM, soybean meal; CAS, casein; DWP, partially delactosed whey powder; SDPP, spray dried porcine plasma; WGM, wheat gluten meal and YMW, yellow meal worm.

Gene set enrichment analysis (GSEA) of differentially expressed genes was performed on all one-to-one comparisons of the possible dietary combinations. The highest number of significantly different gene sets were observed comparing SBM diet to the other diets. The number of significantly regulated gene sets as determined by GSEA is presented in Table 1. When compared to SBM-fed mice, we found 4, 23, 8, and 4 unique differently expressed gene-sets (FDR < 0.05) in CAS, DWP, SDPP and YMW, respectively. These sets of genes mainly correspond to essential cellular processes such as cell cycle, cellular metabolism (anabolic and catabolic) and immune response (Fig 1; S2 Table).

**Table 1:** Differential gene expression and number of enriched gene-sets in ileal mucosa of mice fed experimental diets with indicated protein sources relative to a diet with SBM.

Experimental diet vs SBM	Upregulated gene-sets	Down regulated gene-sets	Significantly enriched gene-sets (FDR < 0.05)*
CAS	559	0	82
DWP	561	0	124
SDPP	591	0	63
WGM	516	0	62
YMW	559	0	98

CAS, casein; DWP, partially delactosed whey powder; SDPP, spray dried porcine plasma; WGM, wheat gluten meal and YMW, yellow meal worm; SBM, soybean meal.

* FDR value was calculated in gene set enrichment analysis (GSEA) for indicated protein sources relative to a diet with SBM.

We identified a large number (thirty-seven) of overlapping gene-sets, which were significantly (FDR < 0.05) differentially expressed across all the experimental diets in comparison with SBM (Fig 1). We calculated an interaction network from these 37 gene-sets and observed down regulation (FDR < 0.001) of a number of immune and metabolic processes in ileal mucosae of the mice kept on SBM-fed diet relative to all other diets (Fig 1). In the next step, we analyzed the degree of distribution (number of interactions) for each node in the Reactome FI network and identified a total of 14 hub genes, which are indicated in Table 2 along with their pathways and biological functions. Strikingly, the mTOR pathway and biological processes related to T cell functioning and antigen presentation are highly represented in Table 2.

**Table 2:** Key or hub genes of the functional network downregulated in mice fed soybean meal (SBM)-based diet.

Hub gene	Degree*	Related pathway	Biological relevance	References
Lck	38	PI3K/AKT/mTOR, MAPK	Selection and maturation of developing T cells.	[27]
Fyn	36	PI3K/AKT/mTOR, MAPK	Regulation of cell growth and adhesion humoral immune response.	[28]
Il2rg	27	PI3K/AKT/mTOR	Critical for intestinal T cell reconstitution	[29]
Cd4	27	TGF β	Augment the early phase of T cell activation. Also found in B cells, macrophages, and granulocytes.	[30]
Cd3e	27	Class I MHC mediated antigen processing and presentation, NF-κB Family Pathway	Antigen recognition by T cell and T cell development.	[31]
Cd3g	25	Class I MHC mediated antigen processing and presentation, PI3K/AKT/mTOR	Antigen recognition by T cell and T cell development.	[32]
Cd3d	24	Class I MHC mediated antigen processing and presentation, PI3K/AKT/mTOR	Antigen recognition by T cell and T cell development.	[33]
Pik3cg	24	PI3K/AKT/mTOR	T cell activation and differentiation. Structural and functional integrity of epithelium.	[34]
Stat1	24	PI3K/AKT/mTOR, Signaling by FGFR	Important for cell viability in response to different cell stimuli and pathogens.	[35]
Lcp2	23	PI3K/AKT/mTOR, Signaling by FGFR	Promotes T cell activation and development as well as mast cell and platelet function.	[36]
Cd8a	21	Class I MHC mediated antigen processing and presentation, TCR signalling	Selection and maturation of developing T cells.	[37]
Ptprc	21	Class I MHC mediated antigen processing and presentation	Regulates cell growth, differentiation, mitosis. Essential regulator of T- and B-cell antigen receptor signaling.	[38]
Zap70	21	Class I MHC mediated antigen processing and presentation, Ras signaling pathway	T cell development and lymphocyte activation.	[39]
Btk	21	PI3K/AKT/mTOR, Signaling by FGFR	B-cell development.	[40]

* Degree is the number of directly connected nodes within functional interaction (FI) network. PI3K/AKT/mTOR, Phosphoinositide 3-kinase/Protein kinase B/mammalian (mechanistic) target of rapamycin; MAPK, mitogen-activated protein kinases; TGF, transforming growth factor; MHC, major histocompatibility complex; NF-κB, nuclear factor kappa-light-chain-enhancer of activated B cells; FGFR, fibroblast growth factor receptors; TCR, T cell receptor.

### Effect of diet on ileal microbiota

**Fig 2:**
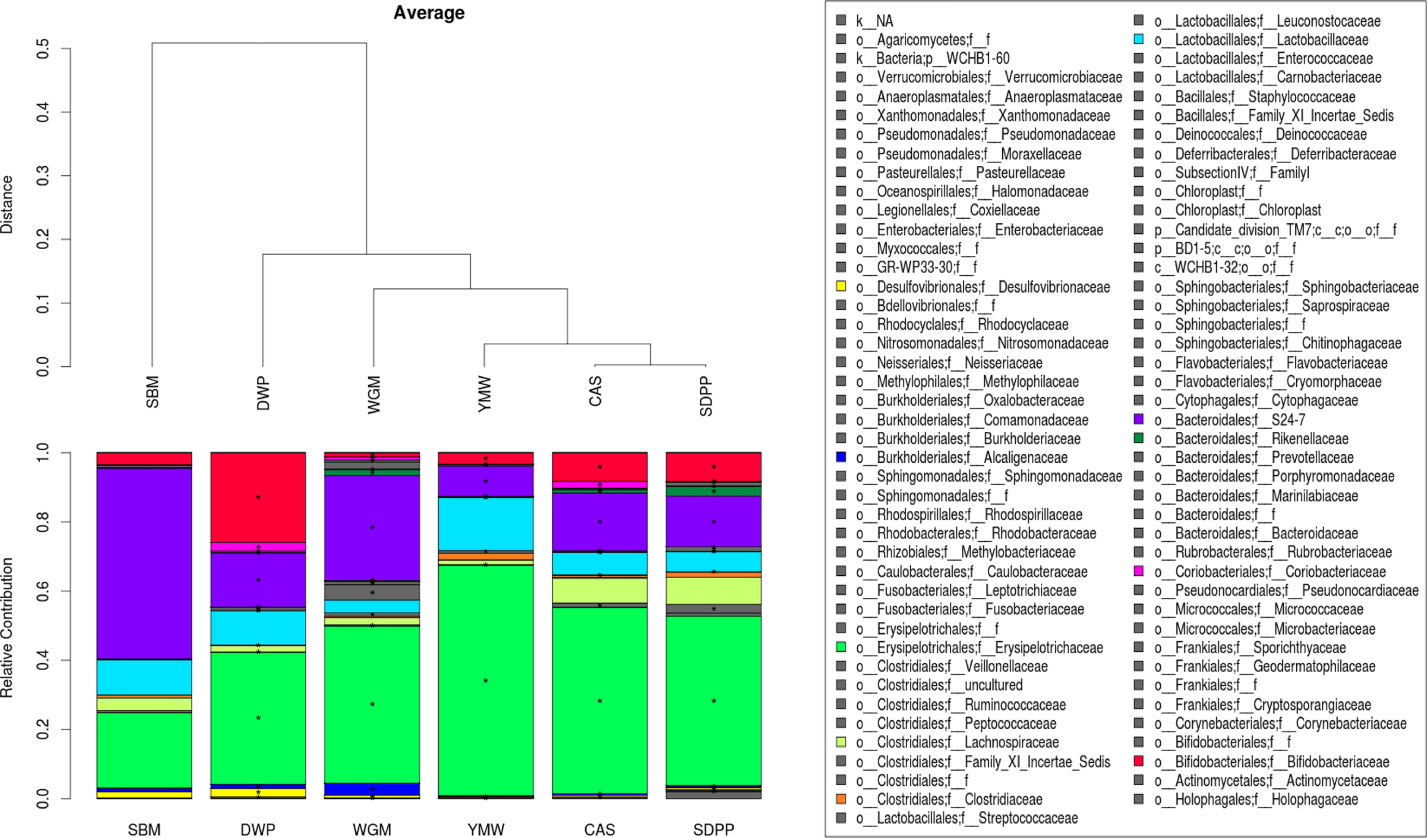
The ileal microbiome composition profiles based on the relative abundance at the family level. Bacterial families are represented with different colors (shown in key). Hierarchical clustering of the microbial family composition is indicated above the composition profile. * Represents significant difference (*P* < 0.05) of microbiota at family level compared to SBM-fed mice. Members of microbial family belonging to ‘others’ are listed in S3 Table. SBM, soybean meal; CAS, casein; DWP, partially delactosed whey powder; SDPP, spray dried porcine plasma; WGM, wheat gluten meal and YMW, yellow meal worm.

**Fig 3:**
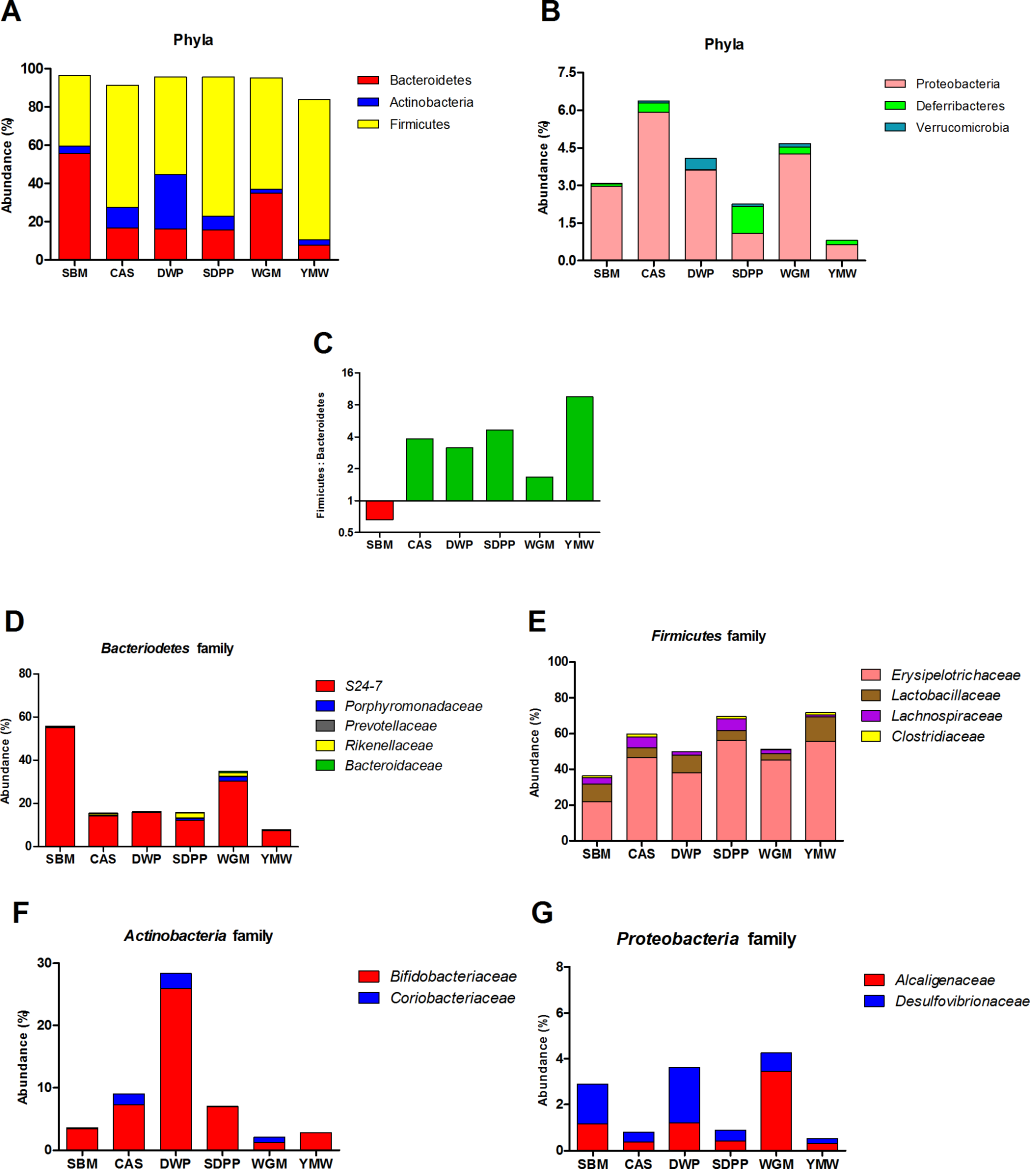
Ileal microbial profiles of mice fed diets containing protein from different sources. (A) and (B) show the microbiota distribution at the phylum level; (C) shows the *Firmicutes/Bacteroidetes* ratio in the various groups of mice; (D-G) show the microbiota distribution at the family level. Bacterial abundance is shown as a percentage of the total 16s RNA gene sequences per group. SBM, soybean meal; CAS, casein; DWP, partially delactosed whey powder; SDPP, spray dried porcine plasma; WGM, wheat gluten meal and YMW, yellow meal worm.

To investigate the dietary effects on intestinal microbiota, we performed 16S rRNA gene sequencing on ileal digesta of six mice per group. Details of sequence reads count and the number operational taxonomic unit (OTU) are given in online supporting material (S3 Fig). Further analysis revealed that the microbiota composition was significantly different among all dietary treatments (Figs 2 and 3). Hierarchical clustering analysis indicated that the microbial profile of SBM-fed mice was significantly different (*P <* 0.05) from the other dietary groups (Fig 2). The protein source had substantial effect on the *Firmicutes* to *Bacteroidetes* ratio of SBM-fed mice was inverted as compared to all the other experimental diets fed mice (Fig 3C). An opposite effect was observed in the YMW-fed group, having an increased *Firmicutes* to *Bacteroidetes* ratio compared to other groups. As these two phyla constitute up to 90% of the microbiota we analyzed their family structure in more detail (Fig 3D and 3E).

Within the *Bacteroidetes*, the proportion of the *S24-7* family was largely increased (Fig 3D) in SBM and WGM-fed mice. The *Erysipelotrichaceae* family (*Firmicutes*) was reduced by 16.4% and 34.5% in the SBM group as compared to the DWP and SDPP groups, respectively (Fig 3E). The *Bifidobacteriaceae* family (*Actinobacteria*) was increased by 22.5%, 3.8% and 3.5% in the DWP, CAS and SDPP groups as compared to the SBM group (Fig 3F). In the phylum *Proteobacteria*, the *Alcaligenaceae* family only increased in the WGM group by 2.3% compared to the SBM group (Fig 3G). The abundance of *Bifidobacteriaceae* was substantially increased in the mice fed de-lactosed whey protein, which may have been due to presence of milk oliogosaccharides which are known to have a prebiotic effect on *Bifidobacterium* species. The proportions of several other families were also affected by the various diets (Figs 2 and 3).

### Protein source influences mTORC1 activity and amount of mTOR protein in ileum tissue

**Fig 4:**
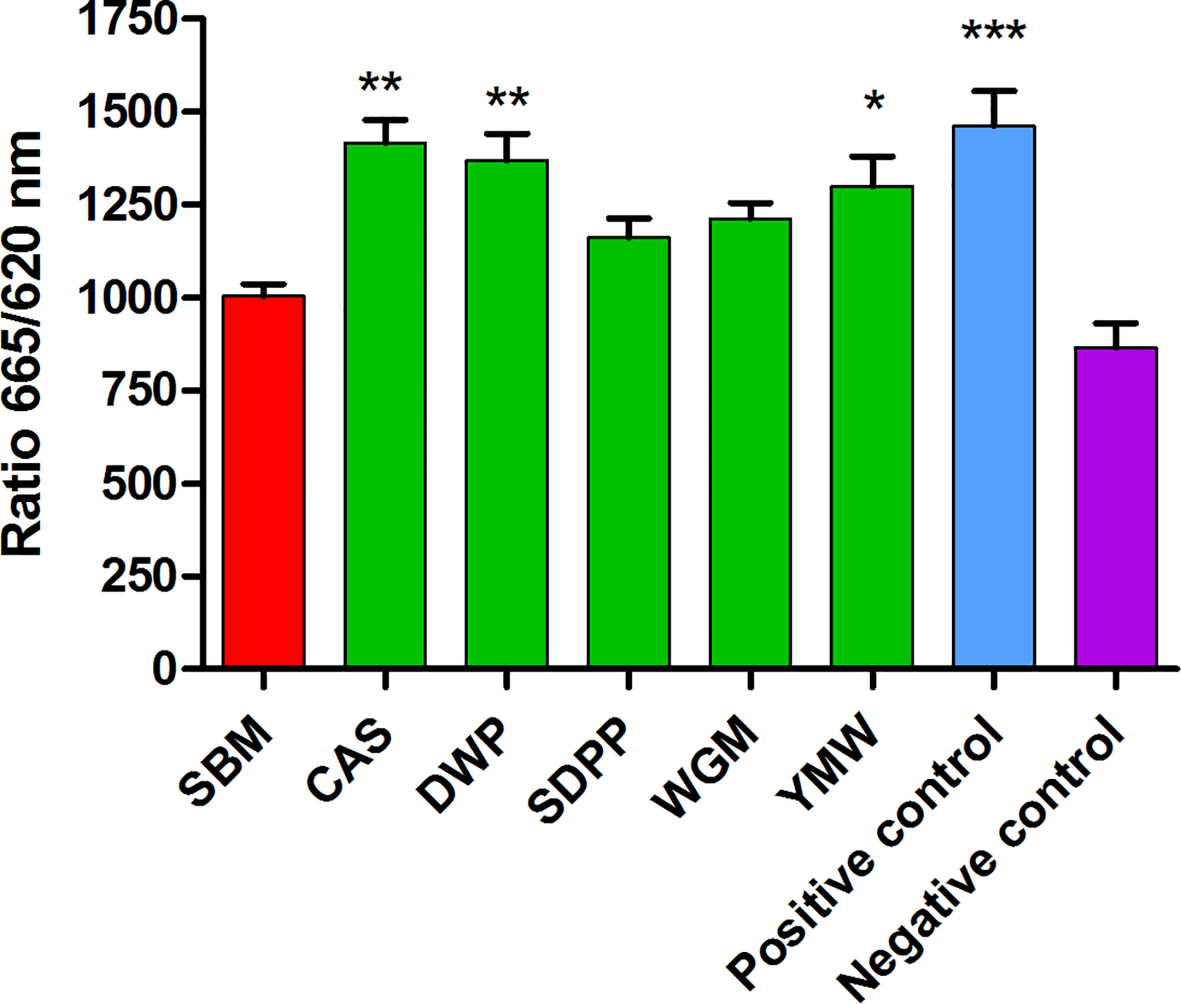
Endogenous levels of phosphorylated mTOR in cells derived from ileal tissue of mice fed with different experimental diets. Bars and whiskers represents means values ± SEM (n = 12, six biological replicates and two technical replicates); *P < 0.05, **P < 0.005, ***P < 0.0005 compared to SBM-fed mice fed. Here, SBM, soybean meal; CAS, casein; SDPP, spray dried porcine plasma; WGM, wheat gluten meal and YMW, yellow meal worm.

Endogenous levels of phosphorylated mTOR in cells derived from ileal tissue of mice fed with SBM diet (the most common source of animal protein) was lowest among all the dietary treatments. The level of phosphorylated mTOR in mice fed with SBM diet was significantly lower compare to CAS-, DWP- and YMW-fed mice (Fig 4). This result suggest that the different protein sources provide different levels nutrients to the epithelial and other cells of the ileum tissue and hence mTOR phosphorylation and activity.

**Fig 5:**
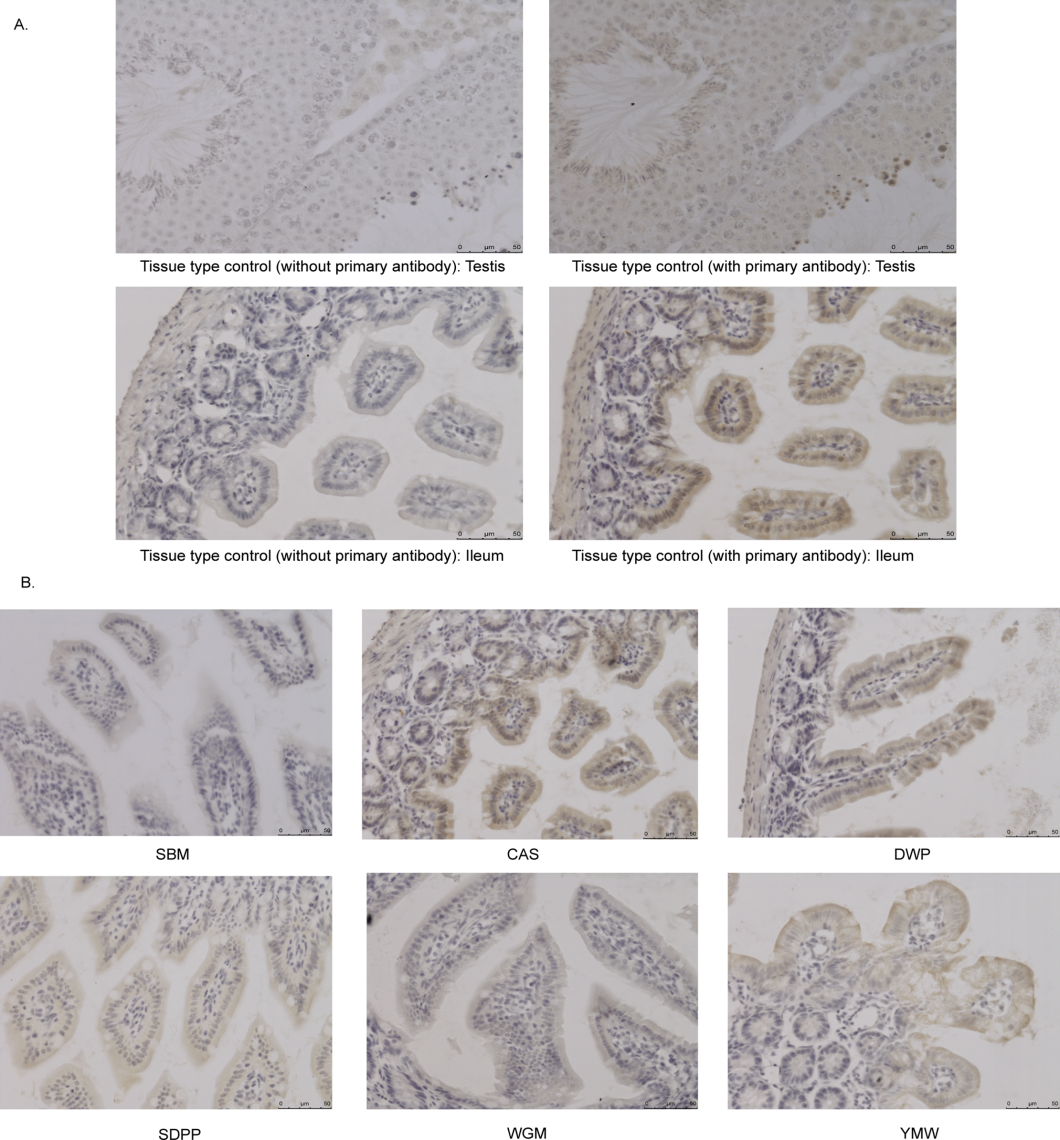
Immunohistochemistry of PFA-fixed paraffin-embedded tissue sections with anti-mTOR antibody in ileal tissue of mice fed with different experimental diets. **(**A) Brown color indicates positive reaction in the image of tissue type control (testis) and a positive ileal tissue section (CAS). No positivity was observed in the same tissue by withholding the primary antibody (i.e. anti-mTOR antibody) during the staining procedure. (B) Immunohistochemistry of PFA-fixed paraffin-embedded ileum sections with anti-mTOR antibody of mice fed with different experimental diets. Brown color in the tissue section indicates positive reaction. SBM, soybean meal; CAS, casein; SDPP, spray dried porcine plasma; WGM, wheat gluten meal and YMW, yellow meal worm. Scale bar: 50 μm.

To investigate whether this might be due to altered expression of mTOR we used antibodies against mTOR to detect the protein in section of ileal tissue from mice fed protein sources diets. Only background levels of staining were seen in sections from mice fed the SBM diet whereas strongest staining of mTOR was observed in the samples with high mTORC1 activity (Fig 5). This suggest that levels of mTOR protein expression correlate with mTOR activity and that protein expression may be increased following activation of mTOR, possibly via an autoregulatory mechanism. mTOR staining was strongest in epithelial cells, suggesting they contribute most to the activity measured in the mTOR activity assay and differential expression mTOR pathway in the transcriptomics data sets.

### Systemic cytokines and chemokines

**Fig 6:**
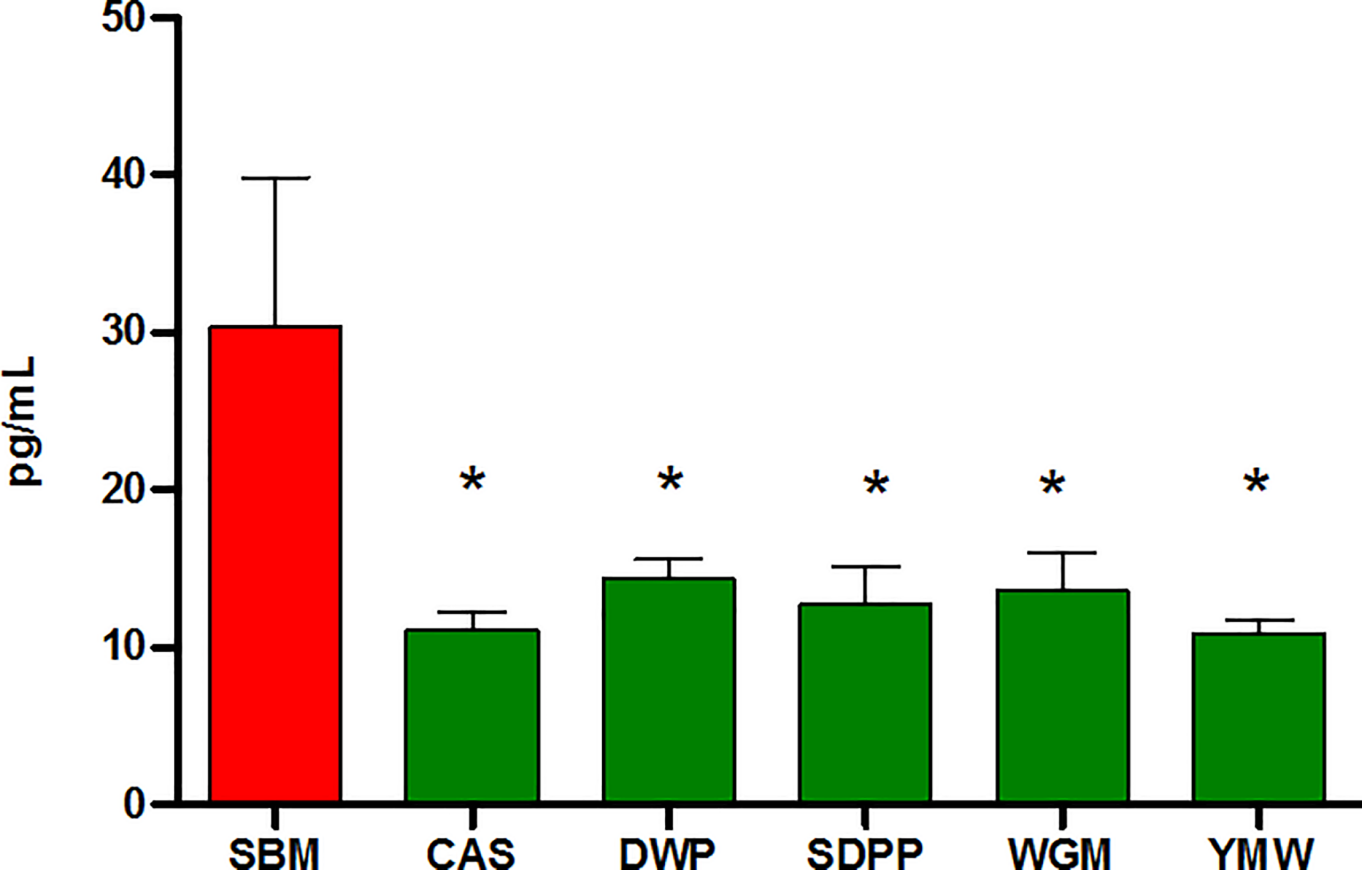
Concentrations of serum granulocyte-colony stimulating factor (G-CSF) of mice fed diets containing different dietary proteins. Bars and whiskers represents means values ± SEM (n = 6), recorded at the end of experiment. *P < 0.05 compared to SBM-fed mice fed. SBM, soybean meal; CAS, casein; DWP, partially delactosed whey powder; SDPP, spray dried porcine plasma; WGM, wheat gluten meal and YMW, yellow meal worm.

To investigate whether the mTOR activity induced by the different diets resulted in any changes in systemic immunity we measured a panel of cytokines and chemokines in the serum of mice from each dietary group (S4 Fig). The serum concentration of EOTAXIN, granulocyte-colony stimulating factor (G-CSF), granulocyte-macrophage colony-stimulating factor (GM-CSF), interferon gamma (IFN-γ), interleukin-2 (IL-2), IL-5, IL-6, IL-12p70, IL-13, monocyte chemotactic and activating factor-1 (MCP-1) and macrophage inflammatory protein-1β (MIP-1β) were significantly different (*P* < 0.05) in at least one of the experimental dietary groups as compared to SBM (S4 Fig). One notable finding was that mice which received SBM recorded two to three times higher (P < 0.05) serum concentrations of G-CSF than groups of mice that received the other experimental diets (Fig 6). Furthermore, significant lower (*P* < 0.05) concentration of GM-CSF, IL-6, IL-13, MCP and MIP-1β were observed in mice fed with WGM based diet compared to SBM.

## Discussion

The protein sources used in the experimental diets varied slightly in energy values and nutrient composition, meaning that differences in the growth and feed intake parameters cannot be directly compared between diets. However, in other respects the diets reflect more accurately the use of these novel protein sources in formulated animal feeds and thus can be expected to reveal diet-related differences in intestinal physiology through transcriptomics and biochemical assays performed on tissue and serum. DWP-fed mice showed a significantly lower feed intake compared to SBM-fed mice and had a lower body weight gain over the experimental period as compared to the other groups. This agrees with previous observations showing that compared to CAS, whey proteins, increase the plasma levels of cholecystokinin and glucagon- like peptide-1 which are linked to satiety [41]. Alternatively, a possible explanation for the reduced body weight in the DWP-fed group could be the high electrolyte balance compare to other experimental diets, related to a high concentration of potassium and high concentrations of other minerals, especially calcium, in the DWP-based diet. Pilvi and colleagues showed that a high calcium diet with whey protein decreased body weight gain in high-fat-fed C57Bl/6J mice [42]. Funkat and colleague reported that mice fed a high fat (60%) diet gain more body weight than mice fed a standard chow diet [43]. We speculate that the higher proportion of fat in YMW (160 g/kg vs. < 90 g/kg for the diets in the other treatments), may have promoted satiety levels via one of the gut lipid sensing system resulting in the observed lower feed intake [44]. This might explain reduced feed intake but normal body weight of mice in the YMW group although, further research would be needed to determine the mechanisms involved.

The ileal transcriptomics data revealed striking differences between groups of mice on the SBM diet and the mice fed diets containing different protein sources. In comparison to the other protein sources, 14 hub genes in gene networks associated with antigen presentation, mTOR signaling and TGFα expression were down-regulated in the mice on the SBM diet. Functionally these genes are of particular relevance to mTOR pathway which has a role in cellular differentiation and proliferation of epithelial cells as well as T cells, B cells and antigen presenting cells in the intestinal lamina propria (Table 2). Activation of mTOR pathway plays a key role in shaping and controlling the effector responses of immune cells associated with innate and adaptive immune responses through coupling these events to intracellular metabolic status and environmental nutrients [45].

To investigate whether the relative down-regulation of gene transcription associated with mTOR regulated cellular processes in SBM-fed mice was linked to reduced mTOR activity, we measured the amount of active phosphorylated mTOR in ileal tissue samples. Indeed, the amounts of phosphorylated mTOR was lowest in mice fed with SBM diet and was significantly lower compared to CAS-, DWP- and YMW-fed mice (Fig 4). Concentrations of amino acids are known to regulate mTOR activity suggesting that the digestibility of the proteins *in vivo* may be influencing the amount of nutrients available to epithelial and other cells of the ileum tissue. Thus, for further understanding of the underlining bio-molecular mechanisms influenced by the dietary protein sources, it is essential to generate *in vivo* data towards kinetics of protein degradation and amino acid digestibility. Understanding of how amino acid levels are influencing mTORC1 activity has only recently come to light and it is possible that other, as yet unknown nutrients, including microbial metabolites may influence mTORC1 activity. Apart from nutrients, various growth factors and cytokines can participate in the regulation of mTOR pathways, which might also be influenced by the diet or indirectly by effect of diet on the microbiota.

Histological detection of mTOR with an anti-mTOR antibody showed least reactivity in the ileum tissue sections of SBM fed mice, which is consistent with which is consistent with the mTOR activity results. As far as we are aware, a correlation between mTOR protein expression and mTOR activity has not, been previously reported, and may be due to an autoregulatory mechanism involving mTOR activation.

As the effects of the SBM diet on mTOR and immunity pathways in the ileal mucosal might be influenced by cross-talk with the microbiota, we analyzed the effects of diet on the ileal microbiota composition by 16S rRNA gene sequencing. Protein source substantially altered the microbiota at phylum and family level, the most striking change being the lowest ratio of *Firmicutes* to *Bacteroidetes* phyla in the SBM-fed group (Fig 3C). *Bacteroidetes* was the most abundant phylum (~55.9%) in the SBM fed group of mice followed by WGM (34.8%) and lowest abundance was seen in the YMW group (7.7%) (Fig 3D). Within the *Bacteroidetes* phylum, the genus *Bacteroides* are known to possess a large number of genes encoding for enzymes involved in the degradation and fermentation of a variety of different carbohydrates [46]. Traditionally, SBM is considered to contain several complex carbohydrates, which includes non-starch polysaccharides (NSP). Thus the NSP from SBM may have caused the blooming of members in *Bacteroidetes* phylum, the most significant being the *S24-7* family.

One consequence of an increased abundance of *Bacteroidetes* could be altered signaling through the pattern recognition receptor nucleotide-binding, oligomerization domain-containing protein-1 (Nod1), which recognizes meso-diaminopimelic acid-containing peptidoglycan found predominantly in Gram-negative bacteria) and Nod2 which is expressed in Paneth cells in the intestinal epithelium detects peptidoglycan structures found in both Gram-positive and Gram-negative bacteria. Peptidoglycan molecules from the gut has been shown to translocate to the circulation and enhance innate functions of neutrophils [47]. Furthermore, Nod1 but not Nod2 has been shown to induce expression of multiple hematopoietic cytokines in bone marrow mesenchymal stromal cells *in vitro*. *In vivo* administration of NOD1 influences the numbers of hematopoietic stem cells and precursors in bone marrow. Interestingly, greater amounts of G-CSF which can mobilize hematopoietic stem cells from the bone marrow into the blood were detected in the serum of SBM-fed group of mice than the mice on other diets. This intriguing observation may be linked to differences in circulating levels of peptidoglycan ligands in mice fed different diets and warrants future investigation into bone marrow hematopoiesis and numbers of circulating granulocytes.

Although the mice fed with YMW based diet consumed significantly less feed compared to mice in the SBM group, the body weight of the mice in both groups were similar. An increase of the *Firmicutes* to *Bacteroides* ratio in YMW fed mice (Fig 3C) is a microbiome signature found in studies on obese mice [48]. An increased *Firmicutes* to *Bacteroides* ratio in mice has been associated with enhanced energy extraction from the diet leading to adiposity and weight gain in weaned and adult mice [49]. The DWP-fed mice showed an increase of microbes belonging to the *Actinobacteria* phylum and mainly driven by bacteria belonging to *Bifidobacteriaceae* family. This is most probably due to the high sugar (lactose and milk oligosaccharide) content of the diet prepared with DWP. It has been shown that infant-associated *Bifidobacterium* spp. efficiently metabolize several low-mass milk oligosaccharides for growth and possess large gene clusters encoding enzymes involved in (milk) oligosaccharide metabolism [50].

SBM is a commonly used source of protein for animal feed and yet results in lower mTOR protein and activity in the small intestinal mucosa, suggesting energy levels may be lower than alternative protein sources. In the future, microbiota transfer experiments in germ-free mice, detailed characterization of immune cell populations in the intestinal lamina propria and studies with highly purified fractions of the SBM component of the diet may help to elucidate the cause of the observed effects and the consequences for health.

## Supporting information

S1 Fig Design of the experiment. (DOCX)

S2 Fig Feed intake (A) and bodyweight (B) of mice fed with different experimental diets.
(DOCX)

S3 Fig The mean number of 16S rRNA sequence reads (A) and the number of OTU (B) counts detected in the ileal samples of mice fed with different experimental diets.
(DOCX)

S4 Fig Concentrations of serum cytokines and chemokines in response to dietary treatment in mice. (DOCX)

S1 Table Ingredient and calculated or analyzed nutrient composition of the experimental diets for mice, as fed basis. (DOCX)

S2 Table Unique significantly expressed gene-sets (FDR <0.05) in ileum of mice fed with different protein sources compared to SBM-fed diet as shown in Fig 1. (DOCX)

S3 Table Members of microbial family belonging to ‘others’ as shown in Fig 2. (DOCX)
