# Supplementary material for: Dietary protein sources differentially affect microbiota, mTOR activity and transcription of mTOR signaling pathways in the small intestine
Source: PLoS One. 2017 Nov 17;12(11):e0188282. doi: 10.1371/journal.pone.0188282 (PMC5693410; doi:10.1371/journal.pone.0188282)
Supplement: S1 Fig — (DOCX) [file pone.0188282.s001.docx]

**Supporting Information**


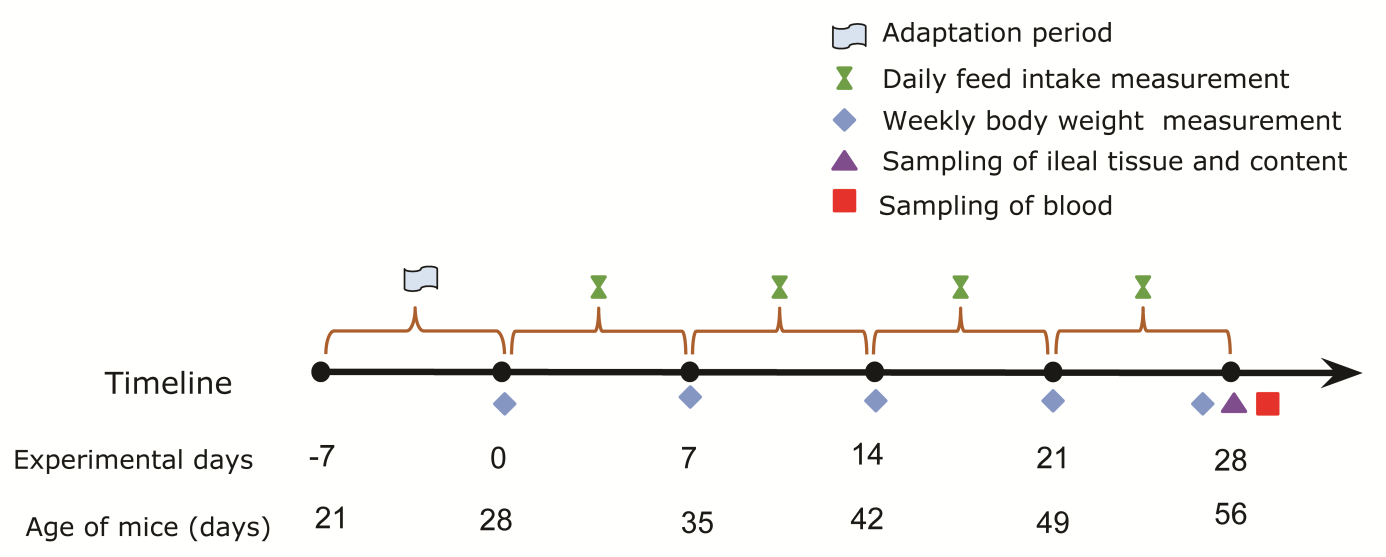


**S1 Fig. Design of the experiment.** The solid black dot in the timeline represents the corresponding experimental days/age of the mice (days). Ileal tissue and its digesta were used for transcriptome and microbiota analysis. Blood was collected for analysis of systemic immune signaling molecules.
