# Supplementary material for: Dietary protein sources differentially affect microbiota, mTOR activity and transcription of mTOR signaling pathways in the small intestine
Source: PLoS One. 2017 Nov 17;12(11):e0188282. doi: 10.1371/journal.pone.0188282 (PMC5693410; doi:10.1371/journal.pone.0188282)
Supplement: S2 Fig — Feed intake (A) and bodyweight (B) of mice fed with different experimental diets. (DOCX) [file pone.0188282.s002.docx]

**Supporting Information**


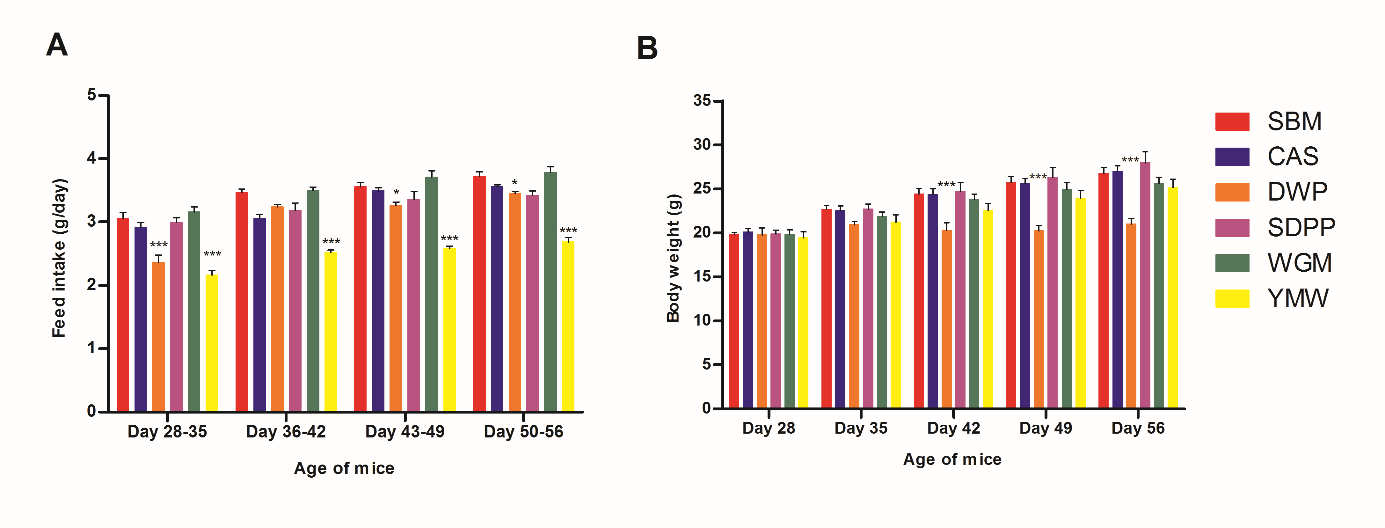
**S2 Fig. Feed intake (A) and bodyweight (B) of mice fed with different experimental diets.** Bars and whiskers represent mean values ± SEM (n = 6), respectively for feed intake (left) and body weight (right) recorded throughout the experimental period. **P <* 0.05, ****P <* 0.001 compared with SBM-fed mice fed. SBM, soybean meal; CAS, casein; DWP, partially delactosed whey powder; SDPP, spray dried porcine plasma; WGM, wheat gluten meal and YMW, yellow meal worm.
