# Supplementary material for: Dietary protein sources differentially affect microbiota, mTOR activity and transcription of mTOR signaling pathways in the small intestine
Source: PLoS One. 2017 Nov 17;12(11):e0188282. doi: 10.1371/journal.pone.0188282 (PMC5693410; doi:10.1371/journal.pone.0188282)
Supplement: S3 Fig — The mean number of 16S rRNA sequence reads (A) and the number of OTU (B) counts detected in the ileal samples of mice fed with different experimental diets. (DOCX) [file pone.0188282.s003.docx]

**Supporting Information**

**
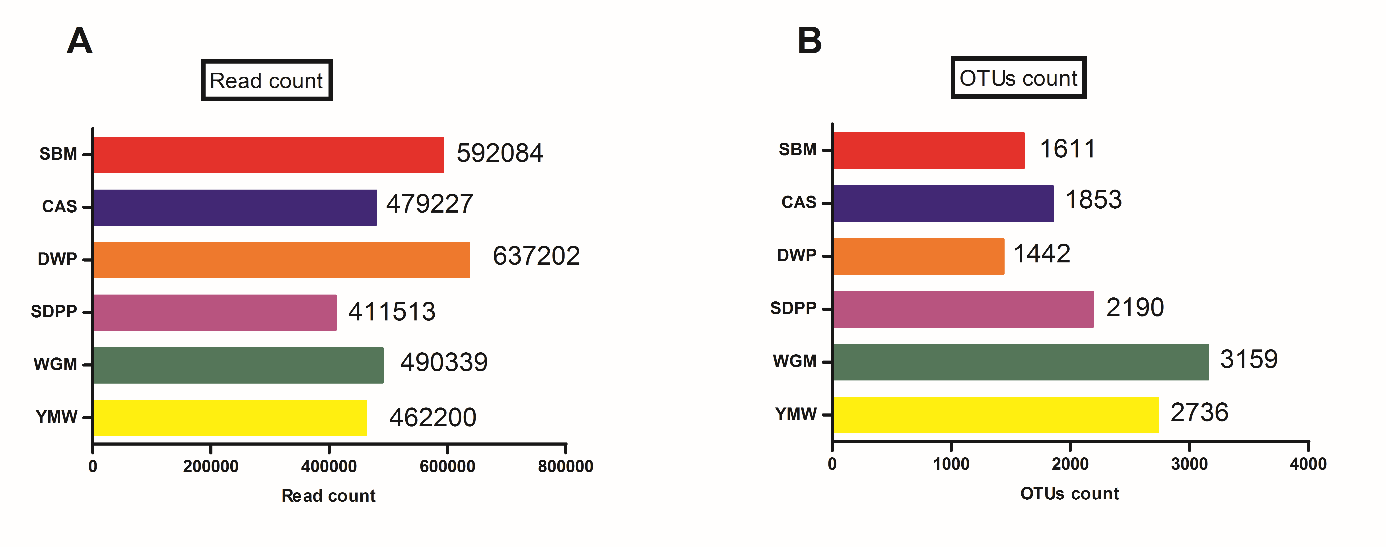
**

**S3 Fig. The mean number of 16S rRNA sequence reads (A) and the number of OTU (B) counts detected in the ileal samples of mice fed with different experimental diets.** SBM, soybean meal; CAS, casein; DWP, partially delactosed whey powder; SDPP, spray dried porcine plasma; WGM, wheat gluten meal and YMW, yellow meal worm.
