# Supplementary material for: Dietary protein sources differentially affect microbiota, mTOR activity and transcription of mTOR signaling pathways in the small intestine
Source: PLoS One. 2017 Nov 17;12(11):e0188282. doi: 10.1371/journal.pone.0188282 (PMC5693410; doi:10.1371/journal.pone.0188282)
Supplement: S4 Fig — (DOCX) [file pone.0188282.s004.docx]

**
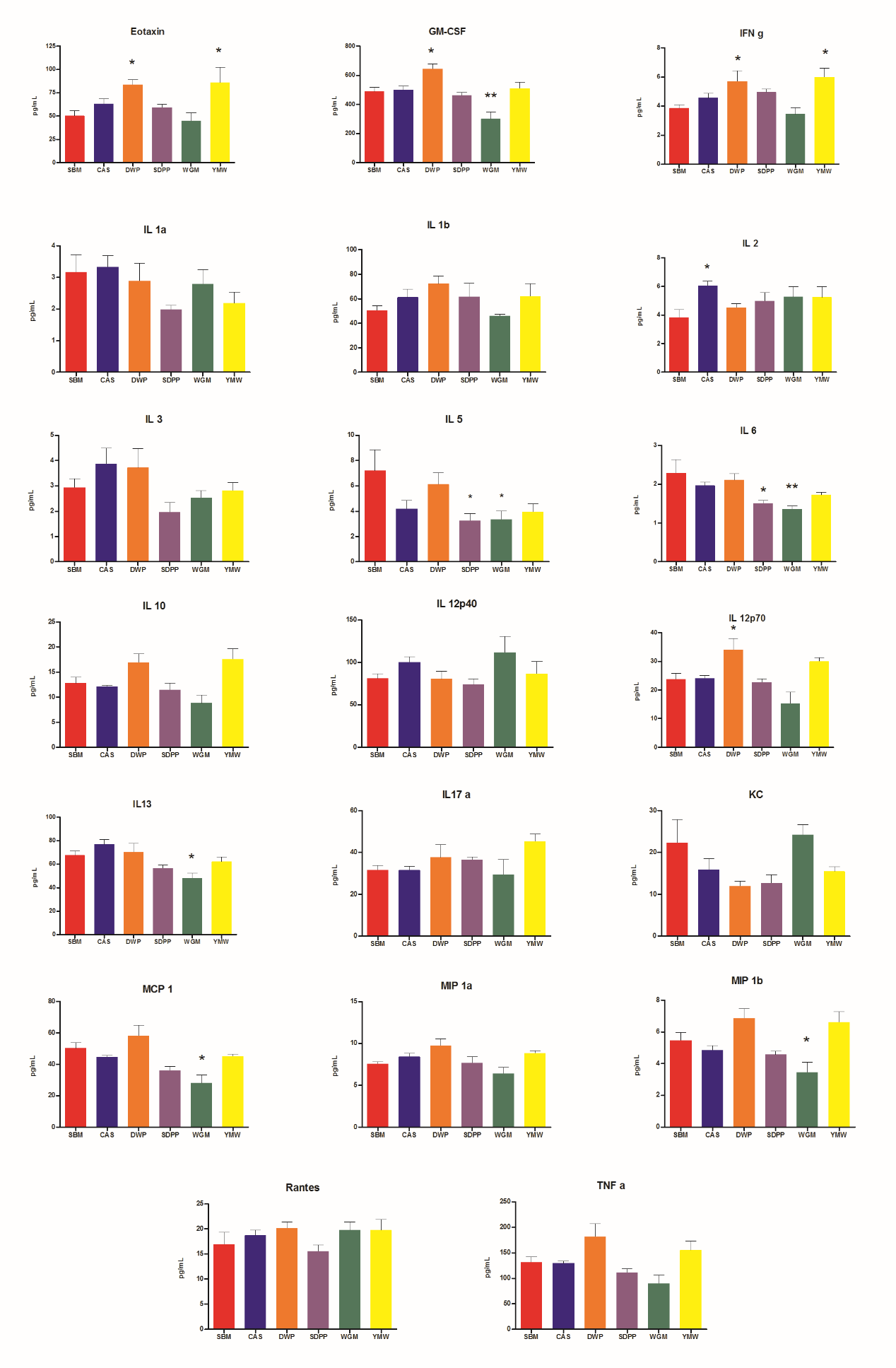
Supporting Information**

**S4 Fig. Concentrations of serum cytokines and chemokines in response to dietary treatment in mice.** Bars and whiskers represent mean values ± SEM (n = 6), for systemic chemokines and cytokines recorded at the end of experiment. **P <* 0.05, ***P <* 0.01, ****P <* 0.001 compared with SBM-fed mice fed. Here, SBM, soybean meal; CAS, casein; DWP, partially delactosed whey powder; SDPP, spray dried porcine plasma; WGM, wheat gluten meal and YMW, yellow meal worm.
