# Supplementary material for: Dietary protein sources differentially affect microbiota, mTOR activity and transcription of mTOR signaling pathways in the small intestine
Source: PLoS One. 2017 Nov 17;12(11):e0188282. doi: 10.1371/journal.pone.0188282 (PMC5693410; doi:10.1371/journal.pone.0188282)
Supplement: S1 Table — (DOCX) [file pone.0188282.s005.docx]

**Supporting Information**

**S1 Table. Ingredient and calculated or analyzed nutrient composition of the experimental diets for mice, as fed basis^1^.**

|  | Diet^2^ | | | | | |
| --- | --- | --- | --- | --- | --- | --- |
| Item | SBM | CAS | DWP | SDPP | WGM | YMW |
| Ingredient, *g/kg* |  |  |  |  |  |  |
| Maize | 297.5 | 297.5 | 297.5 | 297.5 | 297.5 | 297.5 |
| Dextrose | 132 | 132 | 132 | 132 | 132 | 132 |
| Sugar | 100 | 100 | 100 | 100 | 100 | 100 |
| Arbocell | 50 | 50 | 50 | 50 | 50 | 50 |
| Soybean Oil | 70 | 70 | 70 | 70 | 70 | 70 |
| AIN-93G MX | 35 | 35 | 35 | 35 | 35 | 35 |
| AIN-93-VX | 10 | 10 | 10 | 10 | 10 | 10 |
| Choline chloride | 2.5 | 2.5 | 2.5 | 2.5 | 2.5 | 2.5 |
| DL-Methionine | 3 | 3 | 3 | 3 | 3 | 3 |
| Soybean meal | 300 | 0 | 0 | 0 | 0 | 0 |
| Casein | 0.0 | 300 | 0 | 0 | 0 | 0 |
| Delactosed whey powder | 0.0 | 0 | 300 | 0 | 0 | 0 |
| Spray dried plasma protein | 0.0 | 0 | 0 | 300 | 0 | 0 |
| Wheat gluten meal | 0.0 | 0 | 0 | 0 | 300 | 0 |
| Yellow meal worm | 0.0 | 0 | 0 | 0 | 0 | 300 |
| Composition, *g/kg^3^* |  |  |  |  |  |  |
| **Dry matter** | **914** | **957** | **930** | **924** | **917** | **929** |
| **Crude protein** | **153** | **268** | **80** | **238** | **252** | **148** |
| **Ash** | **43** | **29** | **77** | **28** | **47** | **35** |
| Crude fibre | 55 | 44 | 44 | 44 | 46 | 59 |
| **Crude fat** | **76** | **65** | **74** | **87** | **70** | **160** |
| Starch | 251 | 249 | 249 | 249 | 268 | 261 |
| Sugar | 295 | 263 | 403 | 263 | 271 | 263 |
| NSP^4^ | 71 | 8 | 17 | 15 | 2 | 18 |
| **Gross energy, *KJ/g*** | **17** | **19** | **16** | **18** | **18** | **20** |
| Ca | 5.9 | 5.5 | 10.0 | 5.2 | 5.2 | 6.1 |
| P | 3.7 | 3.3 | 6.2 | 1.9 | 2.3 | 4.0 |
| K | 10.4 | 4.0 | 16.5 | 4.5 | 4.0 | 3.6 |
| Na | 1.1 | 1.2 | 5.9 | 8.2 | 1.3 | 1.0 |
| Cl | 1.7 | 2.1 | 10.4 | 12.7 | 1.9 | 1.6 |
| Linoleic acid | 38 | 36 | 36 | 36 | 36 | 36 |
| Electrolyte balance, *Meq/kg* | 266 | 94 | 388 | 115 | 106 | 92 |

^1^Analyzed composition is presented in bold.

^2^Diets: CAS is, DWP is partially delactosed whey powder, SDPP is spray dried porcine plasma, SBM is soybean meal, WGM is wheat gluten meal and YMW is yellow meal worm.

^3^Diets were formulated using data on ingredient nutrient composition and nutrient digestibility coefficients according to the Central Bureau for Livestock Feeding (CVB, Lelystad, the Netherlands).

^4^NSP: Non-starch polysaccharides.
