# Supplementary material for: Dietary protein sources differentially affect microbiota, mTOR activity and transcription of mTOR signaling pathways in the small intestine
Source: PLoS One. 2017 Nov 17;12(11):e0188282. doi: 10.1371/journal.pone.0188282 (PMC5693410; doi:10.1371/journal.pone.0188282)
Supplement: S2 Table — (DOCX) [file pone.0188282.s006.docx]

**Supporting Information**

**S2 Table. Unique significantly expressed gene-sets (FDR <0.05) in ileum of mice fed with different protein sources compared to SBM-fed diet as shown in Fig 1.**

Here, SBM, soybean meal; CAS, casein; DWP, partially delactosed whey powder; SDPP, spray dried porcine plasma; WGM, wheat gluten meal and YMW, yellow meal worm.

| Treatments vs SBM | Serial numbers | Unique gene sets |
| --- | --- | --- |
| CAS |  |  |
|  | 1 | RIBONUCLEOPROTEIN_COMPLEX_BIOGENESIS_AND_ASSEMBLY |
|  | 2 | KEGG_PYRIMIDINE_METABOLISM |
|  | 3 | RIBOSOME_BIOGENESIS_AND_ASSEMBLY |
|  | 4 | RHYTHMIC_PROCESS |
| DWP |  |  |
|  | 1 | PEPTIDYL_TYROSINE_PHOSPHORYLATION |
|  | 2 | DNA_DEPENDENT_DNA_REPLICATION |
|  | 3 | ORGANELLE_LOCALIZATION |
|  | 4 | CELL_CYCLE_GO_0007049 |
|  | 5 | PEPTIDYL_AMINO_ACID_MODIFICATION |
|  | 6 | INTERPHASE |
|  | 7 | PEPTIDYL_TYROSINE_MODIFICATION |
|  | 8 | SMALL_GTPASE_MEDIATED_SIGNAL_TRANSDUCTION |
|  | 9 | POSITIVE_REGULATION_OF_SIGNAL_TRANSDUCTION |
|  | 10 | KEGG_LYSOSOME |
|  | 11 | DNA_METABOLIC_PROCESS |
|  | 12 | CHROMOSOME_ORGANIZATION_AND_BIOGENESIS |
|  | 13 | DNA_REPAIR |
|  | 14 | I_KAPPAB_KINASE_NF_KAPPAB_CASCADE |
|  | 15 | KEGG_NON_SMALL_CELL_LUNG_CANCER |
|  | 16 | INTERPHASE_OF_MITOTIC_CELL_CYCLE |
|  | 17 | REGULATION_OF_SIGNAL_TRANSDUCTION |
|  | 18 | REGULATION_OF_PROGRAMMED_CELL_DEATH |
|  | 19 | KEGG_PROGESTERONE_MEDIATED_OOCYTE_MATURATION |
|  | 20 | REGULATION_OF_MULTICELLULAR_ORGANISMAL_PROCESS |
|  | 21 | REGULATION_OF_APOPTOSIS |
|  | 22 | REGULATION_OF_MAPKKK_CASCADE |
|  | 23 | KEGG_P53_SIGNALING_PATHWAY |
| SDPP |  |  |
|  | 1 | KEGG_PPAR_SIGNALING_PATHWAY |
|  | 2 | KEGG_BIOSYNTHESIS_OF_UNSATURATED_FATTY_ACIDS |
|  | 3 | KEGG_COMPLEMENT_AND_COAGULATION_CASCADES |
|  | 4 | MITOTIC_CELL_CYCLE_CHECKPOINT |
|  | 5 | KEGG_VALINE_LEUCINE_AND_ISOLEUCINE_DEGRADATION |
|  | 6 | KEGG_THYROID_CANCER |
|  | 7 | POSITIVE_REGULATION_OF_T_CELL_ACTIVATION |
|  | 8 | REGULATION_OF_MITOSIS |
| YMW |  |  |
|  | 1 | KEGG_PRION_DISEASES |
|  | 2 | DNA_REPLICATION_INITIATION |
|  | 3 | HOMOPHILIC_CELL_ADHESION |
|  | 4 | REGULATION_OF_DEVELOPMENTAL_PROCESS |
