# Supplementary material for: Dietary protein sources differentially affect microbiota, mTOR activity and transcription of mTOR signaling pathways in the small intestine
Source: PLoS One. 2017 Nov 17;12(11):e0188282. doi: 10.1371/journal.pone.0188282 (PMC5693410; doi:10.1371/journal.pone.0188282)
Supplement: S3 Table — (DOCX) [file pone.0188282.s007.docx]

**Supporting Information**

**S3 Table. Members of microbial family belonging to ‘others’ as shown in Fig 2.**

| Sl No. | Taxa | Sl No. | Taxa |
| --- | --- | --- | --- |
| 1 | o__Holophagales;f__Holophagaceae | 37 | o__Lactobacillales;f__Streptococcaceae |
| 2 | o__Actinomycetales;f__Actinomycetaceae | 38 | o__Clostridiales;f__f |
| 3 | o__Bifidobacteriales;f__f | 39 | o__Clostridiales;f__Family_XI_Incertae_Sedis |
| 4 | o__Corynebacteriales;f__Corynebacteriaceae | 40 | o__Clostridiales;f__Peptococcaceae |
| 5 | o__Frankiales;f__Cryptosporangiaceae | 41 | o__Clostridiales;f__Ruminococcaceae |
| 6 | o__Frankiales;f__f | 42 | o__Clostridiales;f__uncultured |
| 7 | o__Frankiales;f__Geodermatophilaceae | 43 | o__Clostridiales;f__Veillonellaceae |
| 8 | o__Frankiales;f__Sporichthyaceae | 44 | o__Erysipelotrichales;f__f |
| 9 | o__Micrococcales;f__Microbacteriaceae | 45 | o__Fusobacteriales;f__Fusobacteriaceae |
| 10 | o__Micrococcales;f__Micrococcaceae | 46 | o__Fusobacteriales;f__Leptotrichiaceae |
| 11 | o__Pseudonocardiales;f__Pseudonocardiaceae | 47 | o__Caulobacterales;f__Caulobacteraceae |
| 12 | o__Rubrobacterales;f__Rubrobacteriaceae | 48 | o__Rhizobiales;f__Methylobacteriaceae |
| 13 | o__Bacteroidales;f__Bacteroidaceae | 49 | o__Rhodobacterales;f__Rhodobacteraceae |
| 14 | o__Bacteroidales;f__f | 50 | o__Rhodospirillales;f__Rhodospirillaceae |
| 15 | o__Bacteroidales;f__Marinilabiaceae | 51 | o__Sphingomonadales;f__f |
| 16 | o__Bacteroidales;f__Porphyromonadaceae | 52 | o__Sphingomonadales;f__Sphingomonadaceae |
| 17 | o__Bacteroidales;f__Prevotellaceae | 53 | o__Burkholderiales;f__Burkholderiaceae |
| 18 | o__Cytophagales;f__Cytophagaceae | 54 | o__Burkholderiales;f__Comamonadaceae |
| 19 | o__Flavobacteriales;f__Cryomorphaceae | 55 | o__Burkholderiales;f__Oxalobacteraceae |
| 20 | o__Flavobacteriales;f__Flavobacteriaceae | 56 | o__Methylophilales;f__Methylophilaceae |
| 21 | o__Sphingobacteriales;f__Chitinophagaceae | 57 | o__Neisseriales;f__Neisseriaceae |
| 22 | o__Sphingobacteriales;f__f | 58 | o__Nitrosomonadales;f__Nitrosomonadaceae |
| 23 | o__Sphingobacteriales;f__Saprospiraceae | 59 | o__Rhodocyclales;f__Rhodocyclaceae |
| 24 | o__Sphingobacteriales;f__Sphingobacteriaceae | 60 | o__Bdellovibrionales;f__f |
| 25 | c__WCHB1-32;o__o;f__f | 61 | o__GR-WP33-30;f__f |
| 26 | p__BD1-5;c__c;o__o;f__f | 62 | o__Myxococcales;f__f |
| 27 | p__Candidate_division_TM7;c__c;o__o;f__f | 63 | o__Enterobacteriales;f__Enterobacteriaceae |
| 28 | o__Chloroplast;f__Chloroplast | 64 | o__Legionellales;f__Coxiellaceae |
| 29 | o__Chloroplast;f__f | 65 | o__Oceanospirillales;f__Halomonadaceae |
| 30 | o__SubsectionIV;f__FamilyI | 66 | o__Pasteurellales;f__Pasteurellaceae |
| 31 | o__Deferribacterales;f__Deferribacteraceae | 67 | o__Pseudomonadales;f__Moraxellaceae |
| 32 | o__Deinococcales;f__Deinococcaceae | 68 | o__Pseudomonadales;f__Pseudomonadaceae |
| 33 | o__Bacillales;f__Family_XI_Incertae_Sedis | 69 | o__Xanthomonadales;f__Xanthomonadaceae |
| 34 | o__Bacillales;f__Staphylococcaceae | 70 | o__Anaeroplasmatales;f__Anaeroplasmataceae |
| 35 | o__Lactobacillales;f__Carnobacteriaceae | 71 | o__Verrucomicrobiales;f__Verrucomicrobiaceae |
| 36 | o__Lactobacillales;f__Enterococcaceae | 72 | k__Bacteria;p__WCHB1-60 |
| 37 | o__Lactobacillales;f__Leuconostocaceae | 73 | o__Agaricomycetes;f__f |
|  |  | 74 | k__NA |
